# Transcatheter ventricular septal defect closure in children: ten-year experience with multiple devices and long-term outcomes

**DOI:** 10.1186/s43044-026-00753-4

**Published:** 2026-06-01

**Authors:** Gamze Vuran, Murat Muhtar Yılmazer, Mertkan Bilen, Cem Doğan, Yusuf İlker Dur, Ozan Hira, Aykut Özön, Ömer Faruk Gülaştı, Mustafa Karaçelik, Timur Meşe

**Affiliations:** 1 Department of Pediatric Cardiology, University of Health Sciences, Dr. Behçet Uz Pediatric Diseases and Surgery Training and Research Hospital, Izmir, Turkey; 2 Department of Pediatric Cardiac Surgery, University of Health Sciences, Dr. Behçet Uz Pediatric Diseases and Surgery Training and Research Hospital, Izmir, Turkey

**Keywords:** Complications, Long-term outcomes, Occluder devices, Transcatheter closure, Ventricular septal defect

## Abstract

**Background:**

Transcatheter closure has become a widely accepted alternative to surgery for VSD, but long-term pediatric data are limited. This study evaluated procedural success, complications, and long-term outcomes with different occluders.

**Methods:**

We retrospectively analyzed 118 children who underwent attempted transcatheter VSD closure between 2014 and 2024, with clinical, echocardiographic, and electrocardiographic follow-up.

**Results:**

Closure was successful in 110 patients (93%). Failures were due to multifenestrated anatomy, embolization, residual shunt, atrioventricular (AV) block, tricuspid regurgitation, or insufficient aortic rim. Major complications occurred in six patients, including device embolization, progressive aortic regurgitation requiring surgery, persistent nodal rhythm requiring device removal, ventricular perforation, infective endocarditis, and subarachnoid hemorrhage. Minor complications included mild to moderate tricuspid regurgitation (n = 4) and mild aortic regurgitation (n = 5). Arrhythmic events comprised supraventricular tachycardia (n = 2, both resolved), nodal rhythm (n = 1, transient), right bundle branch block (n = 5, persistent in four), and left bundle branch block (n = 1, resolved). Residual shunt was present in 36.4% immediately, declining to 9.8% at follow-up. Larger VSD diameter (OR 1.75, 95% CI 1.14–2.69) and higher body weight (OR 1.07, 95% CI 1.00–1.15) were independent predictors. No late complete AV block, endocarditis, or embolization occurred. Patients treated with Konar-MF were younger and lighter, while Nit-Occlud was associated with higher residual shunt rates (85.7% vs. 33.0%, p = 0.010).

**Conclusion:**

Transcatheter VSD closure in children demonstrated high success, acceptable complication rates, and favorable long-term outcomes. Results were influenced by device choice, defect size, and patient characteristics, highlighting the need for individualized strategies and continued surveillance.

## Introduction

Ventricular septal defects (VSDs) are the most common congenital heart defects, and surgical repair has long been the gold standard of treatment [1]. However, despite high success rates, surgery carries morbidity and mortality risks, including the need for cardiopulmonary bypass, atrioventricular block, residual defects, and valvular insufficiency [2–4]. Transcatheter device closure, offering reduced myocardial injury, lower transfusion requirements, shorter hospital stay, faster recovery, and lower costs, has emerged as a minimally invasive alternative with high success rates [5]. Perimembranous defects, however, remain technically challenging due to their proximity to the conduction system and cardiac valves. Although earlier devices, such as the Amplatzer™ Membranous VSD Occluder, raised concerns of complete atrioventricular (AV) block, newer low-profile and flexible devices (e.g., Amplatzer™ duct occluders, Nit-Occlud® Le VSD coil, Konar-MF™ VSD Occluder) have shown improved safety profiles [6–12]. Nevertheless, long-term outcomes of transcatheter techniques are still limited compared with decades of surgical experience. In this study, we report our ten-year, single-center experience with transcatheter VSD closure using seven different devices and present the long-term outcomes of this evolving procedure.

## Materials and methods

### Study population

This retrospective, single-center study included 118 patients (aged 0–18 years) who underwent transcatheter VSD closure between June 2014 and June 2024 at the Department of Pediatric Cardiology Written informed consent was obtained from the parents or legal guardians of younger children who were not able to provide consent themselves. In accordance with our institutional ethics committee requirements, for children aged 9–18 years, written assent from the child in addition to parental/legal guardian consent was obtained. The study was approved by the University of Health Sciences, İzmir Dr. Behçet Uz Pediatric Diseases and Surgery Training and Research Hospital Clinical Research Ethics Committee. Additionally, artificial intelligence (AI)–assisted technologies were not used in this manuscript or its contents.

Patients with clinically and hemodynamically significant VSDs were eligible for inclusion. Hemodynamic significance was defined as the presence of a left-to-right shunt associated with left ventricular (LV) dilation (left ventricular end-diastolic dimension Z-score ≥ 2), left atrial enlargement (left atrium-to-aortic root ratio > 1.5), or a calculated pulmonary-to-systemic flow ratio (Qp/Qs) > 1.5. In addition to echocardiographic findings, the presence of cardiomegaly on chest radiography, a history of recurrent respiratory infections, symptoms of heart failure, or growth retardation were considered criteria for clinical significance. Patients with a history of infective endocarditis secondary to VSD were also included.

Exclusion criteria were as follows: perimembranous VSD associated with aortic valve prolapse and moderate-to-severe aortic regurgitation (AR), a deficient subaortic rim (< 2 mm) without aneurysmal tissue, severe pulmonary hypertension (pulmonary vascular resistance > 8 Wood units), right-to-left shunting, associated congenital heart defects requiring surgical intervention, or contraindication to antiplatelet or anticoagulant therapy.

### Interventional procedure

All procedures were performed under general anesthesia or deep sedation, with transesophageal (TEE) or transthoracic (TTE) echocardiographic guidance and fluoroscopic monitoring. Prophylactic intravenous antibiotics were administered prior to the procedure. Vascular access was obtained via short femoral venous and arterial punctures, preferably on the right side. Intravenous heparin (100 IU/kg, maximum 5000 IU) was administered to maintain an activated clotting time (ACT) > 200 s. Routine right and left heart catheterizations were performed to assess Qp/Qs. Left ventriculography and aortography were carried out to delineate the VSD anatomy and to evaluate for the presence of aortic valve prolapse or regurgitation. The procedure was conducted using either a retrograde or antegrade approach, in accordance with standard protocols. Before release, device position was confirmed by TTE or TEE. Following device deployment, all patients underwent TTE and left ventriculography, while additional aortography was performed in cases with suspected AR.

The choice of procedural approach evolved over the study period and was influenced by device characteristics/availability, defect anatomy (including the presence of aneurysmal pouch tissue and the relationship of the right ventricular exit to the tricuspid apparatus), and increasing institutional experience. In the early study period, antegrade access with arteriovenous (AV) loop formation was predominantly used, particularly during procedures performed with Amplatzer duct occluders and Cera PMVSD occluders. Since 2018, coinciding with the routine use of the Konar-MF device, the retrograde approach became the primary strategy.

Adequacy of the subaortic rim (defined as ≥ 2 mm) and the presence of aneurysmal pouch tissue were major anatomical determinants in approach selection. In patients with an adequate aortic rim and/or well-developed aneurysmal pouch tissue, retrograde closure was frequently feasible. In cases with a relatively deficient aortic rim but associated aneurysmal pouch tissue, the device was positioned within the pouch to maintain separation from the aortic valve. Prior to release, the delivery cable was advanced further to “tuck” the device into the aneurysmal pouch, thereby increasing the distance from the aortic valve and mitigating the risk of device-related aortic insufficiency. In contrast, patients with a deficient aortic rim without supportive aneurysmal tissue were more commonly managed using an antegrade approach to facilitate controlled positioning. When the antegrade approach was selected, AV-loop formation was preferred over direct venous crossing to provide coaxial alignment and reduce right ventricular manipulations, particularly in defects with restrictive right ventricular exits or complex relationships with the tricuspid chordae.

### Device details and selection

The devices available during the study period included Amplatzer™ Duct Occluder I (ADO I; Abbott, Plymouth, MN, USA), Amplatzer™ Duct Occluder II (ADO II; Abbott, Plymouth, MN, USA), Amplatzer™ Muscular Ventricular Septal Defect (VSD) Occluder (AMO; Abbott, Plymouth, MN, USA), Cera™ Membranous VSD Occluder (symmetric, asymmetric, eccentric; Lifetech Scientific, Shenzhen, China), Cera™ Muscular VSD Occluder (CMO; Lifetech Scientific, Shenzhen, China), Nit-Occlud® Le VSD Coil (PFM Medical, Cologne, Germany), and Konar-MF™ Occluder (Lifetech Scientific, Shenzhen, China).

In the early phase of our experience, device selection depended on defect morphology and availability. ADO I was preferred for perimembranous defects with an adequate aortic rim but right ventricular (RV) exits near tricuspid chordae, where double-disc devices carried a risk of chordal entrapment. Although relatively rigid and potentially associated with aortic regurgitation or conduction block, it was safe in carefully selected cases; sizing was performed with a right disc 1–2 mm larger than the smallest VSD diameter. ADO II, a softer and more mobile device, was limited to small (< 6 mm) defects, although stability was sometimes challenging in aneurysmal VSDs; sizing was based on a waist 1 mm larger than the smallest diameter. Cera™ occluders were similarly stiff and required careful positioning, while AMO and CMO were reserved for muscular VSDs and sized 1–2 mm above the measured diameter. The Nit-Occlud coil was used in seven small aneurysmal perimembranous VSDs, with the distal part at least twice the minimal RV diameter and the LV side 1–2 mm larger than the defect. After 2018, our practice shifted almost entirely to the Konar-MF™ Occluder, whose soft design and low delivery profile enabled use in smaller children. For this device, we selected sizes equal to or only 0–1 mm larger than the RV opening, in contrast to the + 2 mm sizing principle used earlier.

### Follow-up protocol

All patients underwent 12-lead electrocardiography (ECG), TTE, and chest radiography on the first post-procedural day. Follow-up evaluations were performed at 1, 3, 6, and 12 months after the procedure, and annually thereafter. Holter ECG monitoring was performed when clinically indicated. All patients received aspirin therapy (3–5 mg/kg/day) for six months post-procedure.

### Complications

Complications were categorized as major or minor. Major complications were defined as events requiring surgical or additional transcatheter intervention, including device embolization, significant residual shunt, myocardial perforation, severe hemolysis, vessel injury, valvular damage, or persistent complete AV block necessitating permanent pacemaker implantation. Minor complications were defined as non-life-threatening events that resolved spontaneously or with medical management, such as access-site hematomas, transient arrhythmias (including fascicular or bundle branch blocks), minor hemolysis managed medically, blood transfusion due to peri-procedural blood loss, fever, or minor skin reactions.

### Statistical analyses

Statistical analyses were performed using IBM SPSS Statistics version 25.0 (IBM Corp., Armonk, NY, USA). Descriptive statistics were expressed as numbers, percentages, medians, and interquartile ranges. The chi-square test and, when appropriate, Fisher’s exact test were used to analyze associations between categorical variables. For comparisons of continuous variables, the Mann–Whitney U test was applied for two-group analyses and the Kruskal–Wallis test for multiple-group comparisons when data were not normally distributed. Multivariable logistic regression was used to identify factors associated with the development of residual shunts. For regression, we report odds ratios (ORs) with 95% confidence intervals (95% CI). A p-value of < 0.05 was considered statistically significant.

## Results

A total of 118 patients (61 males, 57 females) underwent attempted transcatheter VSD closure. The median age was 6.8 years (minimum–maximum, 0.6–17) and the median weight was 21 kg (minimum–maximum, 5.6–75). Perimembranous VSD was the most frequent defect type (84%), followed by muscular VSD (10%) and postoperative residual VSD (6%). Baseline characteristics are summarized in Table 1.

**Table 1:** The demographic data and characteristics of the cardiac lesions in the patients enrolled in the study

Parameter	Patients^a^ n (%) or Median (IQR)
Gender	
Male	61 (52%)
Age (years)	6.8 (0.6–17)
< 1 year	6 (5)
1–10 years	79 (67)
> 10 years	33 (28)
Weight (kg)	21 (5.6–75)
≤ 10 kg	16 (14)
VSD Types, n (%)	
Native Perimembranous	99 (84)
Native Muscular	12 (10)
Residual Postsurgical	7 (6)
s/p Perimembranous VSD	5
s/p Muscular VSD	1
s/p TOF	1
Concomitant Cardiac Lesions, n	
Small ASD / PFO	8
Mild to Moderate Pulmonary Stenosis (PS)	4
PDA	2
Mild Aortic Regurgitation	5
Mild Mitral Regurgitation	10

*ASD* Atrial Septal Defect, *PDA* Patent Ductus Arteriosus, *PFO* Patent Foramen Ovale, *PS* Pulmonary Stenosis, *VSD* Ventricular Septal Defect, *TOF* Tetralogy of Fallot

^a^The data are presented as median (minimum–maximum) or as number (percentage)

The median procedure and fluoroscopy times were 70 min (range, 30–170) and 15.3 min (range, 6.2–62), respectively. A retrograde approach was used in 53% of procedures. The most frequently used device was the Konar-MF. In two patients, simultaneous patent ductus arteriosus (PDA) closure was performed. Procedural details are presented in Table 2.

**Table 2:** Procedural and device data of the included in the study

Parameter	Value^a^
Angiographic Defect Diameter (mm)	
Left Ventricular Side	6.4 ± 2.8
Right Ventricular Side	4.9 ± 2.2
Procedural Time (min)	70 (30–170)
Fluoroscopy Time (min)	15.3 (6.2–62)
Pulmonary Artery Mean Pressure (mmHg)	16 (10–31)
Vascular Approach, n (%)	
Antegrade	56 (47%)
Retrograde	62 (53%)
Echocardiographic Guidance, n (%)	
TEE	23 (19%)
TTE	87 (74%)
TTE + TEE	8 (7%)
Device Size (mm)	5 (3–12)
Types of occluders used, n	
ADO I	15
ADO II	5
AMO	7
Cera PMVSD Occluder (Total)	12
Symmetric	10
Asymmetric	1
Eccentric	1
Cera Muscular VSD Occluder	3
Nit-Occlud Le VSD Coil	7
Lifetech Konar MF Occluder	69
Occluders Used by VSD Type, n	
Perimembranous (n = 99)	ADO I (14), ADO II (5), Cera PMVSD (12), Nit-Occlud (7), Konar-MF (61)
Muscular (n = 12)	AMO (6), Cera Muscular (3), Konar-MF (3)
Residual (n = 7)	ADO I (1), AMO (1), Konar-MF (5)
Combined Procedure	
PDA Closure	2

*ADO* Amplatzer Duct Occluder, *AMO* Amplatzer Muscular Occluder, *LV* Left Ventricle, *RV* Right Ventricle, *TEE* Transesophageal Echocardiography, *TTE* Transthoracic Echocardiography, *VSD* Ventricular Septal Defect

^a^Values are presented as mean ± SD, median (minimum–maximum), or number (%)

Procedural success was achieved in 110/118 patients (93%). Failure occurred in 8 patients due to large multifenestrated defects (n = 2), device embolization (n = 2), significant residual shunt (n = 1), complete AV block with ST-segment depression (n = 1), moderate-to-severe tricuspid regurgitation (n = 1), and inadequate aortic rim (n = 1). No significant differences were observed between the success and failure groups with respect to age, weight, VSD diameter, device size, or defect type (p > 0.05).

### Complications and follow-up

Major complications developed in six patients (5.1%): device detachment with suspected infective endocarditis (n = 1), subarachnoid hemorrhage 8 h after Nit-Occlud coil implantation (n = 1), persistent nodal rhythm refractory to medical therapy requiring surgical device removal (n = 1), progressive aortic regurgitation requiring surgical explantation and defect repair (n = 1), right ventricular perforation with pericardial effusion requiring surgical repair (n = 1), and immediate device embolization managed by percutaneous retrieval and reimplantation with a larger device (n = 1). In patients who developed major complications, both VSD diameter and device size tended to be larger; however, the results approached but did not reach statistical significance (p ≈ 0.05–0.06).

Minor complications were observed in 26 patients (22%) and included new tricuspid regurgitation (TR) (n = 4), new trivial-mild aortic regurgitation (n = 5), conduction disturbances (n = 9), right ventricular outflow tract stenosis (n = 1), hemolysis (n = 2), femoral artery thrombosis (n = 4), and femoral pseudoaneurysm (n = 1). Major and minor complications are summarized in Table 3.

**Table 3:** Short-/long-term procedure-and device-related complications

Parameter			Value* n
Short-term postprocedural complications	Major	İnfective endocarditis	1
		Nodal arrhythmia	1
		İntracranial bleeding	1
		Aortic regurgitation	1
		Embolization	1
		Right ventricle perforation	1
	Minor	Residual shunt	40
		New tricuspid regurgitation	4
		New trivial-mild aortic regurgitation	5
		RVOT stenosis	1
		Hemolysis	1
		Femoral thrombosis	4
		Femoral pseudoaneurysm	1
		Arrhythmia	9
		LBBB (transient)	1
		RBBB	5
		Nodal ryhtym (transient)	1
		SVT	2
Long-term postprocedural complications	Major	**–**	
	Minor	Residual shunt on the last follow-up	7
		Tricuspid regurgitation	4
		Aortic regurgitation	2
		Arrhythmia	6
		RBBB	4
		Left anterior hemiblock (transient)	1
		Second degree AV block (transient)	1

LBBB: left bundle branch block, RBBB: right bundle branch block, RVOT: right ventricle outflow tract, SVT: supraventricular tachycardia,

*Data are presented as number

**Fig. 1:**
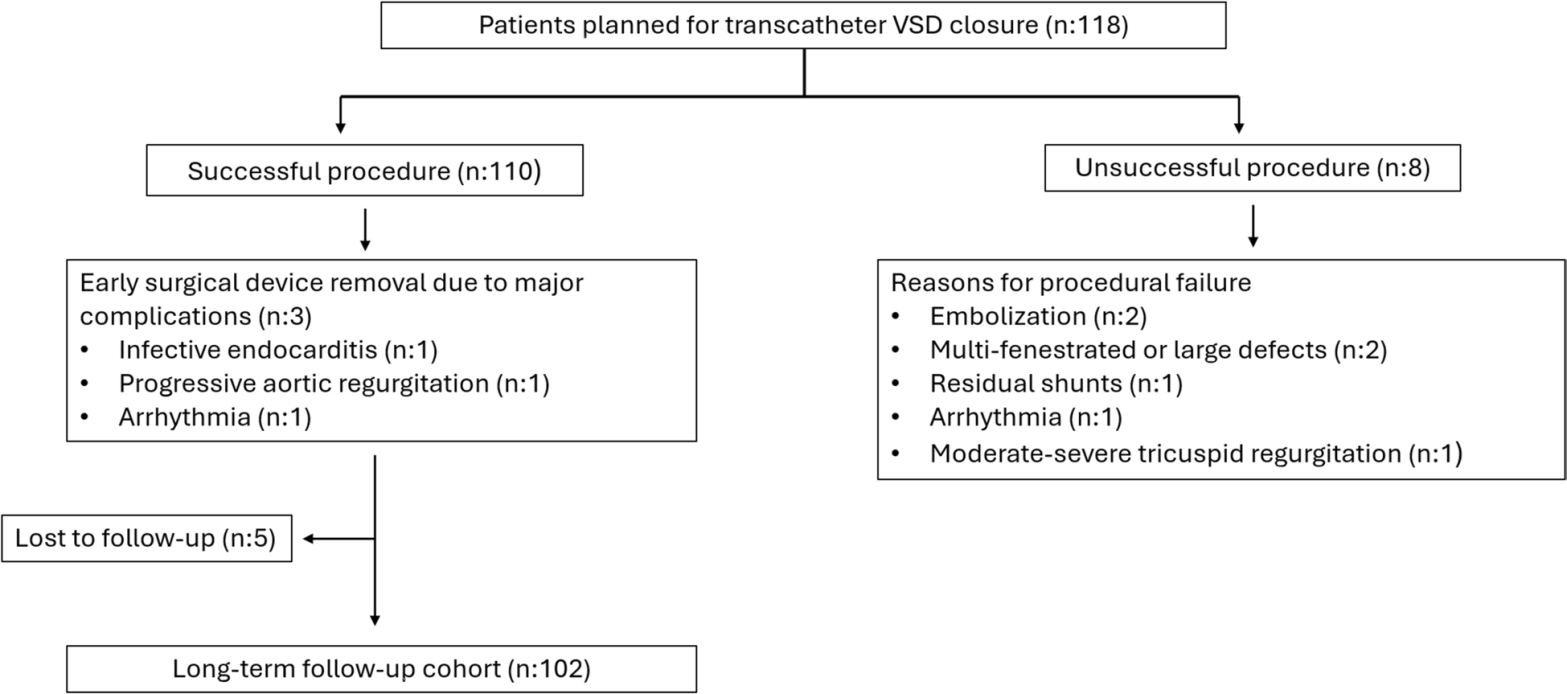
Study flowchart of patients undergoing attempted transcatheter ventricular septal defect closure and long-term follow up availability

Long-term follow-up was available in 102 of the 110 patients with successful transcatheter VSD closure after excluding 3 patients who underwent early surgical device removal because of major complications and 5 patients lost to follow-up (median duration, 6.5 years; range, 1–11) (Fig. 1).Residual shunts were observed in 36.4% immediately after the procedure, decreasing to 9.8% at last follow-up. Patients with residual shunts had significantly larger defects (median 5.1 mm vs. 3.8 mm, p = 0.005) and smaller device–defect differences (0.4 mm vs. 0.95 mm, p = 0.016). In multivariate analysis, both higher body weight (OR 1.07, 95% CI 1.00–1.15, p = 0.042) and larger VSD diameter (OR 1.75, 95% CI 1.14–2.69, p = 0.010) emerged as independent predictors of residual shunt, whereas age, device type, and device size were not significantly associated (Table 4).

**Table 4:** Analysis of factors associated with residual shunt development

Variable	No Residual Shunt (n = 70)	Residual Shunt (n = 40)	p value (Univariate)	OR (95% CI)	p value (Multivariate)
Age (months), median (IQR)	80 (9–201)	84.5 (7–197)	0.84	0.98 (0.96–1.00)	0.059
Weight (kg), median (IQR)	21 (6.5–71)	21 (5.6–75)	0.77	1.07 (1.00–1.15)	0.042*
VSD diameter (mm), median (IQR)	3.8 (2.3–10)	5.1 (2.3–12.6)	0.005*	1.75 (1.14–2.69)	0.010*
Device diameter (mm), median (IQR)	4.0 (3–10)	5.0 (3–12)	0.07	0.73 (0.47–1.12)	0.152
RV device–defect size difference (mm), median (IQR)***	0.95 (0.5–3.5)	0.40 (0.0–3.0)	0.016	–	–
Defect type (PM/Muscular/Residual)	56/9/3	34/3/3	0.55	–	> 0.05
Device type			0.043 (Monte Carlo)**	–	–
Amplatzer Muscular	6	1	n.s	–	–
ADO I	8	4	n.s	–	–
ADO II	3	1	n.s	–	–
CERA PM	8	3	n.s	–	–
Nit-Occlud	1	6	0.010^#^	–	–
Konar-MF	41	25	n.s	–	–
CERA Muscular	3	0	n.s	–	–

*p < 0.05 was considered statistically significant

**Monte Carlo and Fisher exact tests should be interpreted with caution due to small cell counts

***RV device–defect size difference (mm) refers to the difference between the right ventricular disc/waist diameter of the implanted device and the measured VSD diameter

^#^The residual shunt rate was significantly higher in the Nit-Occlud group (85.7%). No statistically significant differences were observed for other device types (n.s.)

Preoperatively, trivial to mild aortic regurgitation was present in 5 patients. One progressed to moderate AR during follow-up, while the others remained stable. Post-procedurally, new AR developed in 5 patients, regressing in 2, persisting as mild in 2, and progressing to mild–moderate in 1. Newly developed TR occurred in 4 patients, persisting as mild in 3 and as mild–moderate in 1 at follow-up. One patient developed right ventricular outflow tract (RVOT) obstruction that resolved within 1 year. Hemolysis was observed in 2 patients: one resolved spontaneously, while the other required blood transfusion. Femoral artery thrombosis occurred in 4 patients, all of whom recovered with short-term anticoagulation. One pseudoaneurysm was successfully treated with thrombin injection.

Holter ECG monitoring was available in 62 of 102 patients (60%). During follow-up, one patient developed transient second-degree type II AV block during sleep in the third year of follow-up, which did not recur. Another patient presented in the fourth year of follow-up with fever and chest pain during the COVID-19 pandemic and was found to have a transient left anterior hemiblock (LAHB).No cases of complete AV block were observed during long-term follow-up.

**Fig. 2:**
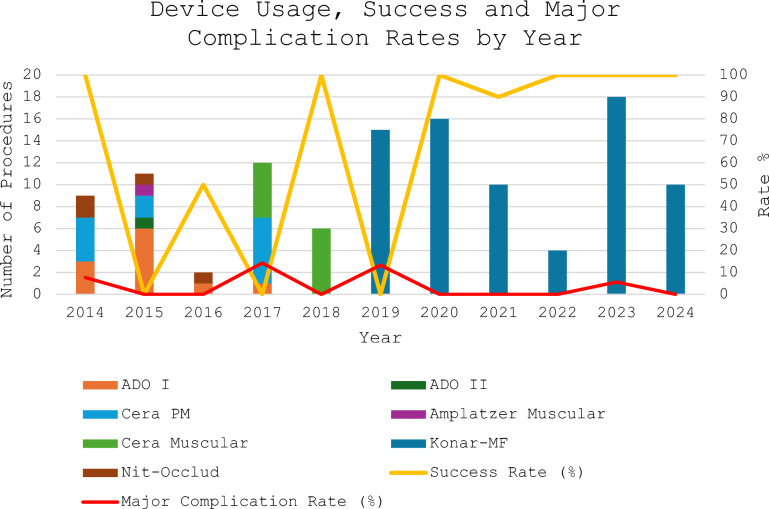
Temporal trends in device selection, procedural success, and major complication rates over the 11-year study period. Multiple device types were used in the early phase (2014–2017), while the Konar-MF occluder became predominant from 2019 onward. Procedural volume and success rates increased progressively, whereas major complications markedly decreased after 2019, reflecting the influence of operator experience and standardization of device choice

Temporal trends in device selection and procedural outcomes are illustrated in Fig. 2.

In the early years (2014–2017), multiple device types including ADO I/II, Cera PM, Amplatzer Muscular, and Nit-Occlud were used, reflecting the initial learning and adaptation phase of the transcatheter closure program. From 2019 onward, the Konar-MF occluder became the predominant device, accompanied by a steady increase in procedural volume and overall procedural success rates exceeding 95%. Major complications were mainly confined to the early years, gradually decreasing to < 5% after 2019. No major complications occurred in the most recent years (2023–2024), suggesting a stabilization of outcomes with growing operator experience and standardization of device choice.

Comparison across devices showed no significant difference in overall procedural success (p = 0.213). Patients treated with Konar-MF were significantly younger and lighter than those in other groups (p = 0.002 for both), and received significantly smaller devices (p < 0.001). Although residual shunts were more frequently observed in patients treated with Konar-MF and Nit-Occlud devices, most were trivial and hemodynamically insignificant. Post hoc analysis showed significantly higher residual shunt rates only in the Nit-Occlud group compared with the others (85.7% vs. 33.0%, p = 0.010) (Table 5).

**Table 5:** Comparison of procedural outcomes, patient characteristics, and follow-up parameters among different device types used for VSD closure

	ADO I^#^ (n = 15)	ADO II ^#^ (n = 5)	AMO^#^ (n = 7)	Cera Mus^#^ (n = 3)	Cera Symm^#^ (n = 12)	Nit-Occlud coil^#^ (n = 7)	Konar- MF^#1^ (n = 69)	p-value^##^
Successful procedure, n	12	4	7	3	11	7	66	0.213
Unsuccessful procedure, n	3	1	0	0	1	0	3	
Age (months), median (range)	136 (74–193)	102 (74–108)	84 (32–197)	73 (53–201)	95 (16–188)	153 (19–169)	63 (7–195)	0.002
Sex, male n (%)	8(53%)	2(40%)	4(57%)	0	6 (46%)	5 (71%)	36(52%)	0.796
Weight (kg), median (range)	45 (18–75)	21 (17.5–31)	21 (13–75)	16 (15–71)	22 (8.2–65)	49 (11.5–65)	18 (5.6–61)	0.002
Device size* (mm), median	6	6	8	8	8	8	4	0.000
Follow-up duration (months), median (range)	121 (87–132)	132 (131–132)	128 (95–131)	101 (81–122)	93 (81–125)	100 (94–100)	50 (14–77)	0.000
Immediate residual shunt, n	4	1	1	0	3	6	25	-
Long-term residual shunt, n	2	0	1	0	0	3	2	-
New tricuspid regurgitation, n	1	0	1	0	0	1	1	-
New aortic regurgitation, n	1	1	0	1	0	0	1	-

*ADO* Amplatzer ductal occluder, *AMO* Amplatzer muscular occluder, *AR* aortic regurgitation, *TR* tricuspid regurgitation

^#^Data are presented as median (range) or number (percentage) where appropriate

^##^A p-value of ≤ 0.05 was considered statistically significant

*Device size refers to waist diameter in mm; for coil and ADO I, the right disc diameter was used

**Statistical comparisons were not performed for groups with fewer than 5 patients

During long-term follow-up, no patient developed late device embolization, infective endocarditis, complete AV block, or thromboembolic events.

## Discussion

This study provides one of the longest follow-up datasets on transcatheter VSD closure in children, encompassing multiple device types over an 10-year period. Our findings confirm a high procedural success rate and an acceptable safety profile, while highlighting the influence of patient selection, device type, and operator experience. Importantly, unlike previous reports with shorter follow-up, we observed no cases of late atrioventricular block, device embolization, or endocarditis, thereby strengthening the evidence for the long-term durability of this approach.

### Residual shunt

Residual shunt remains the most frequent complication after transcatheter closure of VSD. Meta-analyses have reported residual shunt rates of 15–25% in the early post-procedural period, decreasing to 1.5–3% during follow-up [13, 14]. In our cohort, residual shunt was observed in 36.4% of patients post-procedural, declining to 9.8% at the end of follow-up. The relatively higher rate of late residual shunt observed in our cohort may be explained by device heterogeneity, differences in follow-up duration among patients, and the influence of the operator learning curve during the early years of the program. Most shunts were trivial or small, hemodynamically insignificant, and did not require device removal or additional intervention.

Both larger defect size and higher body weight emerged as independent predictors of residual shunt. In our cohort, higher body weight is unlikely to represent a direct causal factor; rather, it may act as a surrogate marker for larger and more complex defects, which are technically more difficult to seal completely. In addition, older and heavier children in our early study period were more frequently treated with earlier-generation devices and during the initial learning phase of the program, which may also have contributed to the observed association. Residual shunts occurred most frequently in patients treated with the Nit-Occlud device, consistent with previous reports linking its design to a higher likelihood of persistent flow [15, 16]. In addition, residual shunts were associated with a smaller device–defect diameter difference, suggesting that slight oversizing may be protective; however, the shift in our sizing strategy with the Konar-MF (from RV defect + 2 mm to + 0–1 mm) did not significantly affect shunt rates, underscoring the importance of tailoring device selection to both defect morphology and occluder design.

### Valve complications

Because of the anatomical proximity of perimembranous VSDs to the aortic and tricuspid valves, valvular complications remain an important concern. In our study, newly developed or progressive regurgitation was uncommon and usually mild, with only one case requiring surgical intervention.

### Tricuspid regurgitation (TR)

Reported incidences of TR after transcatheter closure range from 1.7 to 3.8% [13, 14]. TR was attributable to procedural trauma of the tricuspid apparatus—either catheter/guide-wire interference with the chordae tendineae or impingement of the device’s right disc on the valve. In our series, new TR developed in four patients, mostly mild, which persisted but did not progress in follow-up. To prevent tricuspid regurgitation, we avoided transcatheter closure in VSDs with posterior or inlet extension. During the procedure, the right disc of the device was deployed as close as possible to the interventricular septum to minimize the risk of entrapment of the tricuspid chordae. Continuous monitoring with TEE/TTE was performed, and if chordal entrapment was observed, the device was repositioned.

### Aortic regurgitation (AR)

Development or progression of AR is a leading cause of unplanned surgery after transcatheter VSD closure [18]. Risk factors described in the literature include aortic valve prolapse, inadequate subaortic rim, intracristal defects, oversized devices, and pre-existing AR [17, 18]. Previous studies have reported an incidence of AR ranging from 1 to 5% following transcatheter VSD closure [13, 14]. We did not include patients with aortic valve prolapse, more than mild aortic regurgitation, or a deficient subaortic rim at baseline in our study. In our series, one patient required early surgery for new mild-to-moderate AR due to interference between the LV disc of an oversized device and the aortic valve. This case led us to adopt a strategy of selecting relatively smaller devices. New mild AR developed in five additional patients, of whom only one progressed slightly, while the others remained stable or improved. These results suggest that AR remains uncommon but requires vigilant surveillance.

### Arrhythmia

Complete AV block is a rare but serious complication of transcatheter VSD closure, with meta-analyses reporting an incidence of ~ 1.1%, most within the first post-procedural week [13, 14]. Zhao et al. observed early arrhythmias in 24% of patients, 77.8% of which resolved spontaneously, while Shah et al. reported a rate of 8.5%, most commonly nodal rhythm [19, 20]. Early AV block is attributed to device- or sheath-related trauma or edema, whereas late block is linked to inflammation or fibrosis. In our cohort, no early or late complete AV block was observed. During the procedure, if serious arrhythmias such as complete AV block or left bundle branch block occurred while crossing the defect with a catheter or guide-wire, the intervention was terminated and the patient was considered unsuitable for transcatheter closure. In addition, in recent years we have predominantly used the Konar-MF occluder, which has been reported to exert less mechanical stress on the conduction system due to its softer design. We believe that these two approaches contributed to the absence of AV block in our series.

Nodal rhythm was observed in two patients after device implantation, and both received corticosteroid therapy for one week. One patient reverted to sinus rhythm within the first week, whereas the other had persistent nodal rhythm with chronotropic incompetence and underwent surgical device removal. In the surgically treated case, the arrhythmia was considered to be related to the use of an asymmetric occluder, after which we decided to avoid asymmetric devices in subsequent cases.

### Embolization

Meta-analyses report device embolization in approximately 0.4% of cases, usually occurring immediately or within 24 h after release, and most commonly associated with undersized devices, deficient rims, malposition, or the presence of large left-to-right shunts [13, 14]. In our cohort, embolization occurred in two patients immediately after implantation. In one patient with a residual VSD following repaired tetralogy of Fallot, the device was retrieved with a snare. During repeated attempts, when the occluder was positioned on the surgical patch, the aortic rim was excessively floppy and insufficient to provide stability. Conversely, when the device was placed to compress the patch and rest on native tissue, the aortic disc was found to impinge on the aortic valve. The procedure was therefore abandoned, and the patient was referred for surgical closure. This case illustrates the technical challenges of transcatheter closure in residual defects, where irregular margins, eccentric openings, proximity to the aortic valve, and difficulties in achieving stable device positioning complicate the procedure. In the other patient, the embolized device was retrieved and successfully replaced with a larger occluder, resulting in stable closure.

### Infective endocarditis

Infective endocarditis after transcatheter VSD closure is rare, with only a few cases reported, mostly in the early post-procedural period [21, 22]. One published case described secondary to newly developed aortic regurgitation after transcatheter closure [23]. In our series, infective endocarditis was suspected in one patient who had undergone closure with an Amplatzer muscular VSD occluder in the early post-procedural period, and the device was surgically removed.

### Hemolysis

Hemolysis has been reported in approximately 0.5% of cases following percutaneous VSD closure, occurring more frequently with certain device designs such as the Nit-Occlud [24, 25]. While usually self-limiting, some cases require medical therapy, device removal, or additional implantation. In our series, hemolysis was observed in two patients: in the case closed with a Nit-Occlud device, the hemolysis resolved spontaneously, whereas in the patient treated with an AMO, medical therapy was required.

### Device selection and clinical implications

Over the course of our 11-year transcatheter VSD closure program, our institutional approach to device selection and procedural strategy evolved in parallel with global experience. In the early years, similar to other centers, we used ADO I, ADO II, muscular occluders, Cera, and the Nit-Occlud coil off-label. ADO I was preferred when the RV opening was close to tricuspid chordae to avoid entrapment with double-disc devices. Despite its rigidity and potential for aortic regurgitation or conduction block, it proved safe in carefully chosen patients. ADO II was useful in small (< 6 mm) non-aneurysmal defects but sometimes posed stability challenges in aneurysmal defects, echoing findings from other series. Cera and muscular occluders were effective for larger defects but required strict attention to valve relationships, while the Nit-Occlud coil was reserved for small aneurysmal defects and required technical expertise to ensure stability. During the early phase of the program, heterogeneous device selection was associated with variable success and complication rates. As institutional experience increased and the Konar-MF occluder became the primary choice, procedural success stabilized above 95% and major complication rates markedly declined. These findings underscore the importance of the learning-curve effect and consistent device selection in optimizing procedural outcomes.

Initially, procedures were guided primarily by transesophageal echocardiography, whereas in more recent years transthoracic echocardiography alone has been sufficient for monitoring. For patients treated with ADO I, ADO II, and Cera muscular occluders, our sizing strategy was to select a device approximately 2 mm larger than the measured right ventricular defect diameter. With the introduction of the Konar-MF device, we adopted a more conservative sizing strategy, generally selecting a device with an RV retention diameter equal to or only 1 mm larger than the measured right ventricular defect diameter. Although the use of relatively smaller devices slightly increased the rate of residual shunts, the overall change in sizing strategy did not significantly affect shunt incidence. In our cohort, transcatheter closure was initially performed predominantly in older and larger patients; however, with the introduction of the Konar-MF [26], procedures were increasingly performed in younger and lower-weight children, consistent with recent reports supporting its suitability in smaller patients due to its flexible design and low-profile delivery system. In terms of procedural technique, we initially favored the antegrade approach, but due to the technical challenges of creating an arteriovenous loop and the shorter procedure and fluoroscopy times of the retrograde technique, retrograde closure has become our preferred method. Throughout device deployment, careful assessment of aortic and tricuspid valve function is performed at each step. In cases where serious arrhythmias such as complete AV block or left bundle branch block occur during wire crossing or immediately after device placement, the procedure is terminated, and the patient is considered unsuitable for transcatheter closure.

### Limitations of study

This study has several limitations. First, it was a retrospective, single-center analysis, which may limit the generalizability of the findings. Small sample sizes within certain device subgroups restricted statistical power, particularly for rare complications, and precluded meaningful regression analyses. In our previous study with the Konar-MF occluder, we observed that the learning curve was completed after approximately the first 10 cases, with a subsequent marked reduction in complication rates [26]. Extrapolating this observation, the present study—where seven different occluders were used—likely reflects the impact of both procedural and device-specific learning curves, which substantially influenced the rates of failure and major complications. Furthermore, heterogeneity in VSD type, patient age and weight, and variable follow-up duration further limited subgroup comparisons. Future multicenter, prospective trials with standardized device selection protocols are needed to better identify predictors of complications and to validate long-term outcomes.

## Conclusion

This study comprehensively evaluated the long-term outcomes of transcatheter VSD closure with seven different device types, with follow-up extending up to 11 years in some patients. Our findings confirm that transcatheter VSD closure can be performed with a high success rate and an acceptable safety profile, even in a heterogeneous patient population and across evolving device technologies. Most major and minor complications occurred in the early post-procedural period, whereas during long-term follow-up no cases required surgical or transcatheter re-intervention due to valve insufficiency, embolization, or atrioventricular block. Overall, patients were mainly followed with minor complications only. These findings highlight that careful patient selection, appropriate device choice, and optimal technical performance are crucial to minimize early complications. Moreover, as operator experience increases, the management of early complications becomes more effective, further improving long-term outcomes.

## Data Availability

The datasets used and/or analysed during the current study are not publicly available due to ethical and privacy restrictions related to patient confidentiality, but are available from the corresponding author on reasonable request.

## Abbreviations

AVB: Atrioventricular block
ACT: Activated clotting time
ADO: Amplatzer duct occluder
ADO I: Amplatzer duct occluder I
ADO II: Amplatzer duct occluder II
AMO: Amplatzer muscular occluder
AR: Aortic regurgitation
ASD: Atrial septal defect
CI: Confidence interval
ECG: Electrocardiography
LAHB: Left anterior hemiblock
LV: Left ventricle
OR: Odds ratio
PDA: Patent ductus arteriosus
PFO: Patent foramen ovale
PS: Pulmonary stenosis
Qp/Qs: Pulmonary-to-systemic blood flow ratio
RV: Right ventricle
RVOT: Right ventricular outflow tract
SVT: Supraventricular tachycardia
TEE: Transesophageal echocardiography
TTE: Transthoracic echocardiography
TR: Tricuspid regurgitation
VSD: Ventricular septal defect
